# Long‐Term Selenium‐Yeast Supplementation Does Not Affect Bone Turnover Markers: A Randomized Placebo‐Controlled Trial

**DOI:** 10.1002/jbmr.4703

**Published:** 2022-09-29

**Authors:** Giorgia Perri, Tom R Hill, John C Mathers, Jennifer S Walsh, Fatma Gossiel, Kristian Winther, Jacob Frölich, Lars Folkestad, Søren Cold, Richard Eastell

**Affiliations:** ^1^ Human Nutrition Research Centre, Centre for Healthier Lives, Population Health Sciences Institute Newcastle University Newcastle upon Tyne UK; ^2^ Department of Oncology and Metabolism University of Sheffield Sheffield UK; ^3^ Department of Endocrinology Odense University Hospital Odense Denmark; ^4^ Centre for Diabetes Academic Specialist Centre Stockholm Sweden; ^5^ Department of Molecular Medicine and Surgery Karolinska Institute Solna Sweden; ^6^ Department of Clinical Research University of Southern Denmark Odense Denmark; ^7^ Department of Oncology Odense University Hospital Odense Denmark

**Keywords:** BIOCHEMICAL MARKERS OF BONE TURNOVER, GENERAL POPULATION STUDIES, NUTRITION

## Abstract

Higher selenium status has been associated with lower bone turnover markers (BTM) in epidemiological studies. However, the long‐term impact of selenium supplementation on BTMs has not been studied. We investigated the effects of selenium supplementation on BTMs including osteocalcin (OC), procollagen type I N‐terminal propeptide (PINP), collagen type I cross‐linked C‐telopeptide (CTX), and bone alkaline phosphatase (BALP) in the short (6 months) and long term (5 years). A total of 481 Danish men and women (60–74 years) were randomized to receive placebo‐yeast versus 100, 200, or 300 μg selenium as selenium‐enriched yeast daily for 5 years. Plasma selenium concentration was measured using inductively coupled plasma mass spectrometry, and BTMs were measured in nonfasted samples at baseline, 6 months, and 5 years. Data were analyzed by ANCOVA to investigate the shape of the dose‐response relationships. Covariates included age, body mass index, baseline selenium status, baseline BTM, smoking, alcohol, supplement use, and medication. Plasma selenium concentration (mean 86.5 μg/d at baseline) increased significantly with increasing selenium supplementation to 152.6, 209.1, and 253.7 μg/L after 6 months and remained elevated at 5 years (158.4, 222.4, and 275.9 μg/L for 100, 200, and 300 μg supplemental selenium/d, respectively (*p* < 0.001)). There was no change in plasma selenium concentration in the placebo‐treated group. There was no significant effect of selenium supplementation on OC (6 months *p* = 0.37; 5 years *p* = 0.63), PINP (6 months *p* = 0.37; 5 years *p* = 0.79), CTX (6 months *p* = 0.91; 5 years *p* = 0.58) or BALP (6 months *p* = 0.17; 5 years *p* = 0.53). The relatively replete baseline selenium status in the study participants may explain this lack of effect. Testing in more deficient populations may provide further insights into the impact of selenium supplementation on bone health. © 2022 The Authors. *Journal of Bone and Mineral Research* published by Wiley Periodicals LLC on behalf of American Society for Bone and Mineral Research (ASBMR).

## Introduction

Globally, 0.5 to 1 billion individuals are selenium deficient with blood concentrations below 70 μg/L. Inadequate selenium status in humans impairs the expression of the consortium of selenoproteins that are the biologically active selenium‐containing molecules.^(^ ^1^ ^)^ Selenium intakes vary greatly^(^ ^1^ ^)^; residents of Europe, New Zealand, central Africa, and some parts of China have insufficient selenium intake due to low soil selenium. Among Europeans, selenium intakes declined^(^ ^2^ ^)^ from 60–63 μg/d to 29–39 μg/d from 1970 to 2000.^(^ ^2^, ^3^ ^)^ In Denmark, selenium intake fell from 51 to 42 μg/d during 1990–95,^(^ ^4^ ^)^ and plasma selenium concentration dropped from 42 to 37 μg/L between 1995–2000 and 2002.^(^ ^5^, ^6^ ^)^ However, among older adults, aged 65 to 75 years, intakes increased from the study period by approximately 25 μg/d between 1995–2001 and 2011–2013.^(^ ^7^ ^)^

In humans, selenium deficiency is associated with muscle fatigue, pain, weakness, and increased serum concentration of creatine kinase.^(^ ^8^, ^9^ ^)^ Compared with selenium‐supplemented mice, selenium‐deficient mice had higher concentrations of inflammatory markers and bone resorption markers with poorer bone microarchitecture.^(^ ^10^ ^)^ Similarly, abnormal skeletal growth and poor bone health was observed in selenium‐deficient rats,^(^ ^11^, ^12^, ^13^ ^)^ whereas selenium supplementation improved bone microarchitecture.^(^ ^14^ ^)^

When incorporated into selenoproteins, selenium is important for musculoskeletal function. Most selenoproteins are involved in redox reactions, reducing concentrations of reactive oxygen species (ROS), such as hydrogen peroxide.^(^ ^15^, ^16^ ^)^ Multiple studies have shown an inverse relationship between selenium status and inflammatory molecules, such as interleukin‐6 (IL‐6) and tumor necrosis factor‐α (TNF‐α).^(^ ^17^ ^)^ Both ROS and pro‐inflammatory molecules (e.g. IL‐6, TNF‐α) can initiate bone resorption.^(^ ^18^, ^19^ ^)^ Because selenoproteins are expressed within osteoblasts and osteoclasts,^(^ ^20^ ^)^ the bone formation and resorption cells, respectively, they may regulate bone resorption by moderating oxidative stress through ROS reduction.^(^ ^14^, ^21^, ^22^ ^)^ Higher ROS concentrations increase bone loss through the RANK pathway.^(^ ^23^ ^)^ Participants with lower selenium status have higher concentrations of IL‐6^(^ ^17^, ^24^ ^)^ and supplementation with 200 μg selenium daily for 12 weeks reduced IL‐6 concentration.^(^ ^25^ ^)^ Concentrations of IL‐6 are higher in osteoporotic individuals.^(^ ^23^, ^26^, ^27^ ^)^ In addition, higher selenium concentrations are associated with higher bone mineral density (BMD) and lower bone turnover markers (BTM).^(^ ^28^ ^)^ NHANES data suggested that higher selenium status (mean 131.1 μg/L) in US individuals, especially postmenopausal women, was positively associated with femur BMD.^(^ ^29^ ^)^ Consequently, improving selenium status could be an effective and inexpensive approach to reducing the age‐related decline in bone health. A recent randomized controlled trial (RCT) in 120 postmenopausal women showed that supplementation with sodium selenite for 6 months did not affect bone turnover or BMD.^(^ ^30^ ^)^ Our study aims to extend the findings from this investigation by Walsh and colleagues^(^ ^30^ ^)^ by using both men and women, a larger sample size, and a longer study duration.

This study tested the hypothesis that long‐term supplementation with selenium improves bone health in older adults. We investigated the long‐term effects of selenium supplementation on biomarkers of bone turnover through secondary analysis of data from a 5‐year trial of adults in Denmark who were randomized to supplements providing 100, 200, or 300 μg selenium/d or to a placebo.

## Subjects and Methods

### PRECISE study

Data was obtained from the Danish Prevention of Cancer by Intervention with Selenium (PRECISE): A Pilot Study. The study began in 1998 and ended in 2004 and was organized by the Selenium Centre, Odense University Hospital, Denmark. It aimed to assess the viability for a full randomized controlled trial and hypothesized that selenium supplementation would reduce cancer risk in healthy adults. The trial was registered (ClinicalTrials.gov ID: NCT01819649) and other outcomes from the study have been published.^(^ ^31^, ^32^ ^)^ The regional Data Protection Agency and Scientific Ethical Committees of Vejle and Funen counties approved the study (Journal Number 19980186). The present study is a secondary analysis to determine the effect of selenium supplementation on biomarkers of bone turnover.

### Participants

Using a random sample from the Danish Civil Registration, invitation letters were sent to 2897 men and women aged 60 to 74 years from the County of Funen between November 1998 and June 1999. Of these, 630 accepted the invitation and were screened for inclusion (Supplemental Table S1). A nonfasted blood sample was collected from those meeting the inclusion criteria, and placebo yeast tablets were provided during a 4‐week run‐in phase to determine compliance. At the second visit, participant satisfaction and adherence (>80% of tablets taken using tablet counts^(^ ^31^ ^)^) were assessed. After this, 491 participants met the inclusion and adherence criteria for continuation. All participants provided written informed consent.

### Randomization

**Fig. 1:**
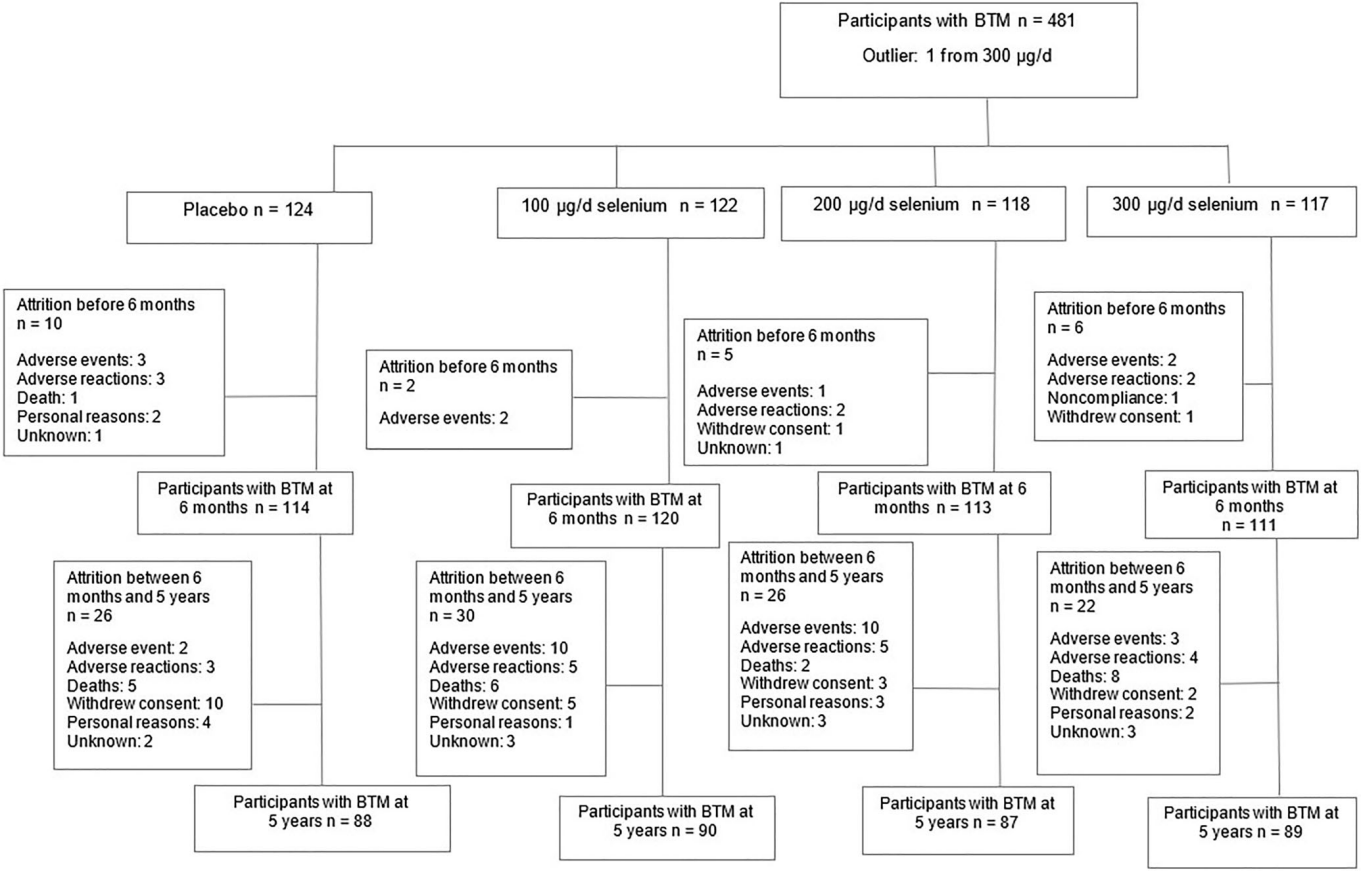
Number of participants with bone turnover marker data at each stage of the study and dropout reasons.

The eligible 491 participants were enrolled in a randomized, double‐blinded, non‐stratified, single‐center, parallel clinical trial with four experimental arms distributed as 1:1:1:1 placebo (yeast tablet; *n* = 126), 100 μg selenium/d (*n* = 124), 200 μg selenium/d (*n* = 122), or 300 μg selenium/d (*n* = 119). There were 482 participants wtih BTM measurements. One participant was removed (see Statistical Analyses below); therefore, participants with BTMs at baseline were *n* = 124, 122, 118, and 117 for placebo, 100, 200, and 300 μg selenium/d, respectively (Fig. 1), giving a total of 481 participants. The study used computer‐generated, blocked, and non‐stratified randomization conducted by the Division of Epidemiology and Biostatistics, Arizona Cancer Centre, University of Arizona. Couples living at the same address were provided with the same intervention supplementation dose for practical reasons, i.e. to prevent mixing of selenium doses. The responsibility of distributing the tablets was placed with pharmacists at Odense University Hospital. Participants, research staff, and investigators were blinded to supplementation doses.^(^ ^31^ ^)^

### Intervention

The selenium was provided as selenium‐enriched yeast (in tablet form) in 100, 200, and 300 μg doses. These doses were suggested to be safe, as the tolerable upper intake level for adversity, set by the Institute of Medicine, is 400 μg/d.^(^ ^33^ ^)^ The SelenoPrecise tablets (prepared by Pharma Nord ApS, Vejle, Denmark) contained 54% to 60% of total selenium as selenomethionine (SeMet) with unknown seleno compounds providing the remainder.^(^ ^34^ ^)^ The placebo was identical to the supplementation tablets and consisted of inactive, spray‐dried baker's yeast (250 μg yeast placebo, 80 μg cellulose, 65 μg dicalcium phosphate, and ≤5 μg of inactive ingredients). Smell and taste were matched by coating all tablets in titanium oxide, and tablets were packaged as 28‐tablet blister packs.

Participant characteristics were determined at baseline. Further evaluations were performed at 6, 12, 18, 24, 36, and 60 months, which included assessment of medical status, tablet count, records of side effects, and the provision of new tablets, as previously described.^(^ ^31^ ^)^

### Biochemical analyses

Nonfasting blood samples were collected at baseline, 6 months, and 5 years. Plasma was prepared and stored at −80°C.

#### Plasma selenium

Total selenium in plasma (μg/L) was measured at baseline, 6 months, and 5 years by LGC Limited using inductively coupled plasma mass spectrometry with external calibration (described in Cold and colleagues^(^ ^31^ ^)^). The selenium concentration for the certified reference standard BCR‐637 was 78.3 (SD 2.7) μg/L (16 independent replicates), indicating good accuracy of the method. High‐selenium concentrations had an intra‐assay coefficient of variation (CV) of 0.5%, whereas low‐selenium concentrations had an intra‐assay CV of 3%. The interassay CV was 3.4%.

#### Bone turnover markers

The rationale for choice of the selected BTMs is described in Supplemental Table S2.^(^ ^35^ ^)^ PINP and CTX were selected because they are the two reference markers recommended by the International Osteoporosis Foundation (IOF) and the International Federation of Clinical Chemistry for inclusion of all studies using bone turnover markers.^(^ ^36^ ^)^ Additionally, they have been associated with selenium and selenoprotein P in the OPUS study, ^(^ ^28^ ^)^ where OC was more closely associated with selenoprotein P than PINP.^(^ ^28^ ^)^ Studies suggest BALP can help identify changes in bone mineralization such as osteomalacia and Paget's disease.^(^ ^37^ ^)^

The BTMs were analyzed in 2017, with study recruitment from 1998–1999 until 2004. Thus, the oldest study samples were 19 years old, and the most recent ones were 13 years old. Studies have suggested that BTMs are stable when stored at −80°C for longer periods of time.^(^ ^38^, ^39^ ^)^ Serum was analyzed from the baseline, 6‐month, and 5‐year time points for N‐MID osteocalcin (OC, measuring the large 1–43 N‐mid and the intact OC), procollagen type I N‐terminal propeptide (PINP, measuring the trimer only), collagen type I cross‐linked C‐telopeptide (CTX), and bone alkaline phosphatase (BALP) at the Bone Biochemistry Laboratory, Department of Oncology and Metabolism, University of Sheffield (England). OC, PINP, CTX, and BALP were measured using the IDS‐iSYS automated immunoassays (Immunodiagnostic Systems, Boldon, UK). The interassay CVs were 5.0%, 7.2%, 6.5%, and 3.5% for OC, PINP, CTX, and BALP, respectively.

### Covariates

Data on covariates were collected during visits with trained research nurses. Body mass index (BMI) was calculated as kg weight/m^2^ height (continuous). Participants were classified into education status using surveys based on time spent in education after public school (0 = no, 1 = 1–3 years, 2 = 3–4 years, 3 = above 4 years). Living status was determined as living alone (0 = no, 1 = yes). Smoking status was determined at baseline (0 = never, 1 = previous, 2 = current). Alcohol intake was reported as standard drinks per week (continuous). Medication usage (thyroid, antiresorptives, glucocorticoids [GC], hormone replacement therapy [HRT]) was classified as a binary variable depending on the medication (0 = no, 1 = yes), with thyroid medication having three categories (0 = none, 1 = levothyroxine, 2 = antithyroid drugs). Supplementation usage (calcium, vitamin D, multivitamins) was classified as a binary variable (0 = no, 1 = yes).

### Statistical analyses

Data were analyzed using the IBM (Armonk, NY, USA) statistical software package version 24.0 (SPSS). A *p* value < 0.05 was considered statistically significant. To determine the normality of the variables, quantile–quantile (QQ) plots were used. Log 10 transformation was applied to all BTM measurements to normalize the data. Participant baseline characteristics are presented according to the supplementation dose. Differences in characteristics across supplementation doses were assessed using chi‐square test (categorical) or Kruskal–Wallis (ordered and non‐normally distributed). Data were analyzed using intention‐to‐treat.

For the main analyses, the shape of the dose‐response relationship between selenium supplementation and each BTM (OC, PINP, CTX, BALP), at each time (6 months and 5 years, respectively), was investigated separately using an ANCOVA with orthogonal polynomials. Covariates included age (continuous), BMI (continuous), baseline selenium status (continuous), baseline BTM (continuous), smoking (binary), alcohol (binary), supplement use (binary; calcium, vitamin D, and multivitamins), and medication (binary; thyroid, systemic and inhaled GC, antiresorptive, and HRT). These covariates were selected based on previous literature showing their effect on bone health.^(^ ^40^ ^)^ Outcomes are reported as estimated marginal means with upper and lower 95% confidence intervals after back transformation.

One participant of the 482 with BTMs was removed from the analyses. This participant's BTM concentrations for OC, PINP, CTX, and BALP were 6‐, 5‐, 7‐, and 2‐fold higher than mean concentrations. The removal of this participant had no significant effect on the main findings or baseline descriptives (data not shown). Sensitivity analyses were undertaken after excluding those participants receiving systemic GC (*n* = 4) because of their potential to influence BTMs.^(^ ^35^, ^39^, ^41^, ^42^ ^)^ A second analysis excluded systemic GC, inhaled GC, antiresorptive, and thyroid medication users (*n* = 53). Although some research suggests inhaled GC have minimal effects on bone,^(^ ^43^ ^)^ a recent article suggested they increased the risk of osteoporosis.^(^ ^44^ ^)^ Thyroid medication has been shown to have a detrimental effect on bone health.^(^ ^45^, ^46^ ^)^ Another sensitivity analysis excluded those using HRT (*n* = 75). A further sensitivity analysis removed supplement users (*n* = 215) because calcium, vitamin D, and multivitamins can influence bone metabolism. A final sensitivity analysis removed those using antiresorptives at baseline, 6 months, and 5 years, as well as those who had fractures, as a proxy to estimate those with osteoporosis (*n* = 14). The analyses were also repeated after categorizing baseline plasma selenium concentration into a binary variable, above and below 70 μg/L, based on evidence that this concentration is required to optimize glutathione peroxidase 3 (GPx) activity.^(^ ^47^ ^)^ Sensitivity analyses are reported in Supplemental Tables S3–S8, respectively.

This was a secondary analysis, sample size was not determined for this particular study. The initial pilot study proposed a sample size of 500 participants.^(^ ^31^ ^)^ The selenium supplemental trial of the effects on bone turnover markers in 120 postmenopausal women conducted by Walsh and colleagues^(^ ^30^ ^)^ was 90% powered to be able to detect 20% between‐group difference in urine N‐terminal cross‐linking telopeptide of type I collagen/creatinine ratio.^(^ ^30^ ^)^ Because our study had similar outcome measures, in 481 participants, we are confident that we have sufficient power to detect any changes in BTMs after selenium supplementation. In addition, a retrospective power analysis was conducted using SAS version 9.4 (SAS Institute, Cary, NC, USA). Least significant changes were set to 20% for OC, 21% for PINP, and 30% for CTX and BALP.^(^ ^48^, ^49^, ^50^ ^)^ These calculations showed that we had >90% power to detect least significant changes in OC, PINP, and BALP at 6 months and 5 years, but only 5% and 16% for CTX at 6 months and 5 years, respectively.

## Results

### Baseline characteristics

Of the 491 participants randomized into the trial, 481 participants had BTM measurements at baseline (Fig. 1). The mean age of participants was 66.2 ± 4.1 years, and there was an almost equal split of male and female participants (52.0 versus 48.0%, *p* = 0.476, respectively) (Table 1). There were significant differences at baseline between supplementation groups for living status (*p* = 0.047), calcium supplementation (*p* = 0.028), and vitamin D supplementation (*p* = 0.020), but, otherwise, the supplementation groups were well matched (Table 1). Overall mean plasma selenium concentration at baseline was 86.5 ± 16.2 μg/d and did not differ between groups (*p* = 0.190; Table 2). Across all supplementation groups, 12% of participants had evidence of selenium inadequacy (plasma concentration < 70 μg/L). Mean ± (SD) baseline concentrations of OC, PINP, CTX, and BALP were 18.7 ± 8.5, 42.7 ± 18.1, 0.20 ± 0.22, and 15.7 ± 5.7 μg/L, respectively (Table 3). Over the 5 years of study, 127 participants were lost to follow‐up, leaving 354 for the full study duration (Fig. 1). There were no differences between supplementation groups in loss to follow‐up (*p* = 0.847) or reasons for dropout (*p* = 0.816, data not shown). However, participants who dropped out were more likely to have lower selenium status at 6 months (*p* = 0.009) but not at baseline. There were no other significant differences in baseline characteristics between participants who dropped out and those who did not (Supplemental Table S9). BTM concentrations did not differ significantly between those who dropped out and those who remained in the study (Supplemental Table S10).

**Table 1:** Baseline Characteristics of Participants with Bone Turnover Marker Measurements, Randomized to Selenium Supplementation (0–300 μg/d)

Characteristic	All participants	Selenium dosage (μg/d)				*p* Value
		0	100	200	300	
	*N* = 481	*n* = 124	*n* = 122	*n* = 118	*n* = 117	
Male, *n* (%)	250 (52.0)	59 (23.6)	69 (27.6)	64 (25.6)	58 (23.3)	0.476
Female, *n* (%)	231 (48.0)	65 (28.1)	53 (22.9)	54 (23.4)	59 (25.5)	
Age (years), mean (SD)	66.16 (4.10)	65.42 (3.8)	66.49 (4.2)	66.32 (4.3)	66.45 (4.1)	0.155
BMI (kg/m^2^) (SD)	26.83 (4.02)	26.51 (4.1)	27.01 (3.8)	27.24 (4.3)	26.51 (4.0)	0.320
Height (m), mean (SD)	1.69 (0.09)	1.68 (0.09)	1.69 (0.08)	1.70 (0.09)	1.69 (0.08)	0.538
Weight (kg), mean (SD)	76.87 (13.5)	75.41 (12.6)	76.84 (11.6)	79.21 (15.4)	75.83 (14.3)	0.255
Alcohol units per week, mean (SD)	7.30 (7.5)	7.75 (8.4)	7.75 (8.0)	7.25 (7.3)	6.37 (6.3)	0.741
Smokers, *n* (%)						
Never	158 (32.8)	36 (22.8)	40 (25.3)	39 (24.7)	43 (27.2)	0.590
Previous	178 (37.0)	45 (25.3)	47 (26.4)	49 (27.5)	37 (20.8)	
Present	145 (30.1)	43 (29.7)	35 (24.1)	30 (20.7)	37 (25.5)	
Education, *n* (%)						
No further education	134 (28.7)	38 (28.4)	41 (30.6)	30 (22.4)	25 (18.7)	0.267
1–3 years	76 (16.3)	13 (17.1)	19 (25.0)	21 (27.6)	23 (30.3)	
3‐4 years	212 (45.4)	57 (26.9)	44 (20.8)	53 (25.0)	58 (27.4)	
>4 years	45 (9.6)	12 (26.7)	14 (31.1)	10 (22.2)	9 (20.0)	
Live alone, *n* (%)						
No	400 (85.7)	94 (23.5)	107 (26.8)	99 (24.8)	100 (25.2)	0.047
Yes	67 (14.3)	26 (38.8)	11 (16.4)	15 (22.4)	15 (22.4)	
Thyroid medication, *n* (%)						
None	467 (97.1)	122 (26.1)	118 (25.3)	113 (24.2)	114 (24.4)	0.659
LT4	11 (2.3)	2 (18.2)	3 (27.3)	3 (27.3)	3 (27.3)	
ATD	3 (0.6)	0 (0.0)	1 (33.3)	2 (66.7)	0 (0.0)	
Inhaled GC, *n* (%)						
No	450 (96.4)	118 (26.2)	111 (24.7)	110 (24.4)	111 (24.7)	0.374
Yes	17 (3.6)	2 (11.8)	7 (41.2)	4 (23.5)	4 (23.5)	
Systemic GC, *n* (%)						
No	475 (99.2)	122 (25.7)	121 (25.5)	117 (24.6)	115 (24.2)	0.595
Yes	4 (0.8)	2 (50.0)	1 (25.0)	0 (0.0)	1 (25.0)	
Antiresorptives, *n* (%)						
No	461 (98.7)	118 (25.6)	117 (25.4)	113 (24.5)	113 (24.7)	0.884
Yes	6 (1.3)	2 (33.3)	1 (16.7)	1 (16.7)	2 (33.3)	
HRT, *n* (%)						
No	392 (83.9)	98 (25.0)	98 (25.0)	99 (25.3)	97 (24.7)	0.740
Yes	75 (16.1)	22 (29.3)	20 (26.7)	15 (20.0)	18 (24.0)	
Calcium, *n* (%)						
No	419 (89.7)	110 (26.3)	112 (26.7)	95 (22.7)	102 (24.3)	0.028
Yes	48 (10.3)	10 (20.8)	6 (12.5)	19 (39.6)	13 (27.1)	
Vitamin D, *n* (%)						
No	442 (94.6)	115 (26.0)	117 (26.5)	103 (23.3)	107 (24.2)	0.020
Yes	25 (5.4)	5 (20.0)	1 (4.0)	11 (44.0)	8 (32.0)	
Multivitamin, *n* (%)						
No	325 (69.6)	90 (27.7)	86 (26.5)	70 (21.5)	79 (24.3)	0.116
Yes	142 (30.4)	30 (21.1)	32 (22.5)	44 (31.0)	36 (25.4)	
Dropout, *n* (%)						
6 months	23 (18.1)	10 (43.5)	2 (8.7)	5 (21.7)	6 (26.1)	0.133
5 years	104 (81.9)	26 (25.0)	30 (28.8)	26 (25.0)	22 (21.2)	

ATD = antithyroid drugs; BMI = body mass index; HRT = hormone replacement therapy; inhaled GC = inhaled glucocorticoid; LT4 = levothyroxine; SD = standard deviation; systemic GC = systemic glucocorticoid.

Age, alcohol *n* = 481; height, weight, BMI *n* = 478; smoking, sex, thyroid medication *n* = 480; BMI, weight, height *n* = 478; systemic GC *n* = 479; education, live alone, inhaled GC, antiresorptives, HRT and supplement users *n* = 467.

**Table 2:** Plasma Selenium Concentration at Baseline, 6‐Month, and 5‐Year Measurements for Participants With Bone Turnover Marker Measurements Randomized to Selenium Supplementation (0–300 μg/d)

Selenium status (μg/L), mean (SD)	All participants	Selenium dosage (μg/d)				*p* Value
		0	100	200	300	
Baseline (*n* = 479)	86.5 (16.2)	85.9 (15.3)	87.8 (16.2)	88.3 (16.4)	84.0 (16.9)	0.190
6 months (*n* = 426)	174.1 (72.4)	85.2 (14.3)	152.6 (23.7)	209.1 (42.2)	253.7 (53.7)	<0.001
5 years (*n* = 349)	185.6 (85.4)	87.5 (24.1)	158.4 (28.4)	222.4 (41.1)	275.9 (78.9)	<0.001

Baseline selenium status *n* = 479: 124, 122, 117, 116; 6 months *n* = 426: 106, 112, 106, 102; 5 years *n* = 349: 88, 88, 86, 87 for 0–300 μg/d selenium, respectively.

**Table 3:** Plasma Concentration of Bone Turnover Markers at Baseline for Participants Randomized to Selenium Supplementation (0–300 μg/d)

Bone turnover (μg/L), mean (SD)	All participants (*N* = 481)	Selenium dosage (μg/d)				*p* Value
		0	100	200	300	
OC	18.7 (8.5)	19.1 (8.2)	18.3 (8.3)	18.0 (8.8)	19.3 (8.5)	0.321
PINP	42.7 (18.1)	43.4 (19.3)	43.0 (16.4)	41.6 (19.8)	42.7 (16.5)	0.629
CTX	0.20 (0.22)	0.21 (0.13)	0.18 (0.11)	0.22 (0.40)	0.21 (0.14)	0.167
BALP	15.7 (5.7)	15.3 (5.5)	15.8 (5.7)	15.6 (5.4)	16.0 (6.2)	0.901

BALP = bone alkaline phosphatase; CTX = collagen type 1 cross‐linked C‐telopeptide; OC = osteocalcin; PINP = procollagen type 1 N‐terminal propeptide; SD = standard deviation.

OC and PINP *n* = 481: 124, 122, 118, 117; CTX *n* = 459: 118, 117, 110, 114; BALP *n* = 479: 124, 121, 117, 117 for 0–300 μg/d selenium, respectively.

### Effects of increasing doses of supplemental selenium on plasma selenium concentration

Over the 5 years of the study, mean ± (SD) plasma selenium concentration in the placebo group remained unchanged (85.9 ± 15.3, 85.2 ± 14.3, and 87.5 ± 24.1 μg/L at baseline, 6 months, and 5 years, respectively, *p* = 0.190; Table 2). In contrast, at 6 months, plasma selenium concentration increased significantly in a dose‐dependent manner with increasing supplemental selenium to reach 152.6, 209.1, and 253.7 μg/L for selenium doses 100–300 μg/d, respectively, and remained elevated at 5 years (Table 2).

### Effects of increasing dose of supplemental selenium on concentrations of bone turnover markers

Concentrations of each of the four BTMs in serum at 6 months and at 5 years were similar to those at baseline and there was no evidence that selenium supplementation altered any of the BTMs at either time point (Table 4). These findings remained robust in sensitivity analyses after excluding (i) users of systemic GC, (ii) combined users of systemic GC, inhaled GC, antiresorptives, and thyroid medication, (iii) users of HRT, (iv) users of nutritional supplements, (v) users of antiresorptives at baseline, 6 months, and 5 years, and those having fractures (Supplemental Tables S3–S7). When analyses were limited to participants with plasma selenium status below 70 μg/L, supplementation had a marginal, significant effect on CTX concentrations at 5 years (Supplemental Table S8).

**Table 4:** Estimated Marginal Means From ANCOVA of Bone Turnover Markers by Supplementation Group at 6 Months and 5 Years

Bone turnover (μg/L), mean (CI)	Selenium dosage (μg)				*p* Value
	0	100	200	300	
OC 6 months	17.1 (16.3–17.9)	16.7 (16.0–17.5)	17.5 (16.6–18.3)	16.5 (15.7–17.3)	0.373
OC 5 years	16.9 (15.6–18.2)	17.1 (15.8–18.6)	17.1 (15.7–18.6)	16.0 (14.8–17.3)	0.630
PINP 6 months	38.7 (36.8–40.8)	36.6 (34.7–38.5)	38.4 (36.4–40.5)	38.6 (36.6–40.8)	0.370
PINP 5 years	39.5 (36.1–43.3)	40.0 (36.5–43.8)	39.4 (35.8–43.3)	37.6 (34.3–41.1)	0.793
CTX 6 months	0.16 (0.14–0.17)	0.16 (0.14–0.17)	0.16 (0.15–0.18)	0.15 (0.14–0.17)	0.910
CTX 5 years	0.16 (0.14–0.18)	0.17 (0.15–0.19)	0.16 (0.14–0.18)	0.15 (0.14–0.19)	0.582
BALP 6 months	14.4 (13.8–14.9)	13.7 (13.2–14.3)	13.7 (13.2–14.3)	14.3 (13.7–14.9)	0.170
BALP 5 years	14.0 (13.2–14.8)	14.8 (14.0–15.7)	14.5 (13.6–15.4)	14.5 (13.7–15.3)	0.525

1 2 3

### Adverse events

Adverse events for the full study have been reported and consisted of grooved nails, hair loss, and skin reactions.^(^ ^31^, ^32^ ^)^ During the 5 years, 22 (4.6%) participants with BTM measurements died and 57 (11.9%) withdrew due to nonfatal adverse events and reactions (Fig. 1) with no significant differences between supplementation groups (*p* = 0.727).

## Discussion

In this RCT of selenium supplementation in older Danish adults, plasma selenium concentration did not have a significant effect on BTMs.

These results are consistent with findings from a RCT of selenium supplementation for 6 months in older women in the UK.^(^ ^30^ ^)^ That study recruited 120 osteoporotic and osteopenic postmenopausal women (aged 55 to 83 years) with a baseline plasma selenium concentration (79.4 μg/L) similar to our present study (86.5 μg/L) and found no effect of selenium supplementation on any of the measured BTMs.^(^ ^30^ ^)^

The plasma selenium concentration for optimal bone health is not known with certainty. However, if it is assumed that the concentration that is adequate for wider aspects of health (≥70 μg/L^(^ ^47^ ^)^) is also adequate for bone, then it is likely that only a small minority of PRECISE study participants (12.1%) had potential to benefit from selenium supplementation. Studies that have observed responses to selenium, such as cancer prevalence or BMD, are usually in populations with suboptimal intakes or status, whereas replete populations are less responsive or nonresponsive.^(^ ^51^, ^52^, ^53^ ^)^ The relatively high baseline selenium concentration (≥70 μg/L^(^ ^47^ ^)^) in the present study may explain the lack of response in BTMs to selenium supplementation.

Specific forms of selenium that have been used for supplementation studies differ in their bioavailability.^(^ ^54^ ^)^ For example, sodium selenite was used by Walsh and colleagues,^(^ ^30^ ^)^ whereas selenium yeast was used in our study and the Nutritional Prevention of Cancer Study.^(^ ^55^ ^)^ Our supplement contained 54% to 60% SeMet^(^ ^34^ ^)^; studies suggest that the bioavailability of SeMet is more than 90%, whereas it is around 50% for the inorganic forms, selenite and selenate.^(^ ^56^ ^)^ Selenium yeast is one of the most bioavailable forms of selenium with high efficiency in increasing selenoenzyme activity.^(^ ^57^, ^58^ ^)^ Therefore, it is unlikely that the lack of effect of selenium supplementation on BTMs observed in the present study was attributable to low selenium bioavailability. Indeed, supplementation raised plasma selenium concentration in a dose‐dependent manner (*p* > 0.001).

Among the strengths of our study, to our knowledge, this is the first, long‐term (5 years), large‐scale RCT exploring the effects of selenium supplementation on BTMs in older men and women. There was a large dose‐dependent increase in plasma selenium concentration with supplementation, suggesting compliance was good. This was gauged by the linear increase in selenium concentration with an increasing daily dose at 6 months, which was maintained at 5 years. In contrast, the plasma selenium concentration remained similar to baseline in the placebo group. Plasma selenium concentration is a robust indicator of selenium status^(^ ^59^ ^)^ and correlates well with recent intakes of organic selenium.^(^ ^60^ ^)^ The use of BTMs can help determine metabolic imbalances within bone, fracture risk, and detect nonresponders to treatment. Using a range of biomarkers allowed us to overcome some of the individual limitations of each BTM to provide a more representative finding, as well as using markers suggested by the IOF.^(^ ^35^, ^36^ ^)^

A limitation of our study was the use of nonfasted blood samples as feeding can decrease BTMs.^(^ ^61^ ^)^ Circadian rhythm can also affect BTMs, especially markers of bone resorption, for which concentrations are highest in the morning.^(^ ^62^, ^63^, ^64^, ^65^ ^)^ Consequently, the use of nonfasted samples may increase the variance in BTM measurements, but this is likely to be similar for all supplementation groups. We have no information on dietary intakes of other nutrients that influence bone health, such as calcium and vitamin D,^(^ ^40^, ^66^ ^)^ although we were able to adjust for supplementation with calcium, vitamin D, and multivitamins. We do not have data on BMI change through the study, but mean values at baseline were similar for all supplement groups, so this is unlikely to have been a confounder. Our participants were aged 60 to 74 years at baseline and so we are unable to generalize our results to older populations among whom osteoporosis and micronutrient deficiencies are more likely.^(^ ^40^, ^67^ ^)^ Future studies exploring the effects of selenium supplementation on bone health should consider including older people with lower baseline selenium status and/or those with inflammatory conditions who are at greater risk of osteoporosis and who may be more responsive to selenium supplementation.

This was the first long‐term (5 years) RCT exploring the effects of selenium supplementation on BTMs in healthy older adults. Selenium supplementation did not have any significant effect on BTMs. We cannot rule out the potential of selenium supplementation to improve bone health in adults with lower selenium status and/or poorer bone health at baseline.

## Disclosures

RE received consultancy funding from IDS, Sandoz, Samsung, Haoma Medica, CL Bio, Biocon, Coherus, and Takeda; meeting presentations for Pharmacosmos, Alexion, and Amgen; and grant funding from Roche, Pharmacosmos, and Alexion. JSW received speaker's honoraria from Eli Lilly and Sandoz, grant funding from Alexion, donation of drug from Eli Lilly for clinical studies, and consulting fees from Mereo Biopharma. All other authors state that they have no conflicts of interest.

## Author Contributions

**Giorgia Perri:** Formal analysis; writing – original draft; writing – review and editing. **Tom R Hill:** Supervision; writing – review and editing. **John C Mathers:** Supervision; writing – review and editing. **Jennifer S Walsh:** Conceptualization; data curation; funding acquisition; writing – review and editing. **Fatma Gossiel:** Conceptualization; data curation; investigation; writing – review and editing. **Kristian Winther:** Conceptualization; data curation; investigation; supervision; writing – review and editing. **Jacob Frølich:** Conceptualization; data curation; writing – review and editing. **Lars Folkestad:** Conceptualization; data curation; writing – review and editing. **Søren Cold:** Investigation; writing – review and editing. **Richard Eastell:** Conceptualization; data curation; funding acquisition; writing – review and editing.

### Peer Review

The peer review history for this article is available at https://publons.com/publon/10.1002/jbmr.4703.

## Supporting information


**Appendix S1.** Supplementary Information.

## Data Availability

The data that support the findings of this study are not openly available.

## Footnotes

1 CI = confidence intervals; OC = osteocalcin; PINP = procollagen type 1 N‐terminal propeptide; CTX = collagen type 1 cross‐linked C‐telopeptide; BALP = bone alkaline phosphatase.

2 Upper and lower 95% confidence intervals are displayed in parentheses.

3 Covariates in the ANCOVA included age (continuous), body mass index (continuous), baseline selenium status (continuous), baseline bone turnover marker (continuous), smoking (binary), alcohol (binary), supplement use (binary) (calcium, vitamin D, and multivitamins) and medication (binary) (thyroid, inhaled and systemic glucocorticoid [GC], antiresorptives, hormone replacement therapy [HRT]). OC 6 months *n* = 404: 107, 105, 98, 94; OC 5 years *n* = 328: 84, 84, 76, 84; PINP 6 months *n* = 403: 106, 105, 98, 94; PINP 5 years *n* = 328: 84, 84, 76, 84; CTX 6 months *n* = 378: 99, 98, 93, 88; CTX 5 years *n* = 299: 74, 77, 73, 75; BALP 6 months *n* = 402: 106, 104, 98, 94; BALP 5 years *n* = 328: 84, 83, 77, 84 participants for 0–300 μg/d selenium, respectively.
