# Supplementary material for: Long‐Term Selenium‐Yeast Supplementation Does Not Affect Bone Turnover Markers: A Randomized Placebo‐Controlled Trial
Source: J Bone Miner Res. 2022 Sep 29;37(11):2165–73. doi: 10.1002/jbmr.4703 (PMC10087503; doi:10.1002/jbmr.4703)
Supplement: Supplementary file 1 — Appendix S1. Supplementary Information. [file JBMR-37-2165-s001.pdf]

Supplemental Material

Supplemental Table 1: Inclusion and exclusion criteria.

Inclusion	Exclusion
60-74 years	A Southwest Oncology Group performance status score >1, indicating impairment in general well-being and activities of daily life
Any sex	Active liver or kidney disease (alanine aminotransferase, alkaline phosphatase, bilirubin or urea two standard deviations above the normal reference range)
Healthy volunteers	Previous diagnosis of cancer (excluding non-melanoma skin cancer)
World Health Organization performance status 0 or 1	Diagnosed HIV infection
No active liver- or kidney disease (serum alanine aminotransferase (ALAT), alkalic phosphatase, bilirubin, creatinine or urea within 2 S.D of laboratory reference range)	Receiving immunosuppressive therapy
No previous cancer diagnosis	Unable to understand written and spoken information
No known HIV-infection	Receiving ≥50 μg/d of selenium supplements in the previous 6 months (by patient report).
Participant must understand oral and written information	
Participant must not use selenium supplementation of above 50 μg/d	
Participant must give written consent prior to inclusion	

Sensitivity Analyses

After removal of users of systemic GC (n = 4), systemic or inhaled GC, antiresorptive or thyroid medication (n = 53), supplements (n = 215) or AR at baseline, 6 months and 5 years or with fractures (n=14), selenium supplementation did not have a significant effect on any of the BTMs at either time point (Supplemental Tables 3,4,6,7). After removal of HRT users (n = 75), 200 μg supplementation had a significant effect on BALP at 6 months ($P = 0.041$, $\eta2 = 0.013$), but not overall dosage (Supplemental Table 5). A final sensitivity analysis was performed that was limited to participants with a baseline plasma selenium status below 70 μg/L (12% of the population). Concentrations of CTX at 5 years significantly differed in a linear trend ($P = 0.011$) between dosages in those participants with a baseline selenium status below 70 μg/L ($P = 0.045$, $\eta2 = 0.036$) where 200 and 300 μg dosages led to a linear reduction in CTX compared to the placebo ($P = 0.011$, $\eta2 = 0.282$ and $P = 0.020$, $\eta2 = 0.243$, for each dose, respectively) (Supplemental Table 8).

Supplemental Table 2: Bone turnover markers and evidence for feasibility to estimate bone health (adapted from Burch et al. 2014 [35]).

Bone turnover marker	Function	Evidence	Reference
**OC**	Formation<br>Characterises osteoblastic activity<br>Bone specific marker	More closely associated with SePP than PINP<br><br>Increases with growth and fracture recovery<br><br>Low within person variability<br><br>Lower in those using corticosteroids	[28,35]
**PINP**	Formation<br>Characterises osteoblastic activity<br>Precursor of collagen type 1 made by osteoblasts	Associated with selenium and SePP<br><br>Recommended by IOF and IFCC<br><br>More sensitive than other BTMs and less affected by feeding status and diurnal variation	[28, 35, 36]
**CTX**	Resorption<br>Characterises osteoclastic activity and hydrolysis of collagen	Associated with selenium and SePP<br><br>Recommended by IOF and IFCC<br><br>More responsive that other BTMs with bisphosphonates	[28, 35, 36]
**BALP**	Formation<br>Characterises osteoblastic activity<br>Marker of mineralisation<br>Plays role by promoting hydroxyapatite crystal growth<br>Corresponds to high osteoblastic activity	Review of usefulness of BALP; low variability, useful for follow up studies<br><br>Low biological and diurnal variation<br><br>Increases during fracture recovery, Paget’s disease, rickets, osteocalcin, osteoporosis, vascular calcification, chronic kidney disease	[35, 37]

OC: osteocalcin; PINP: procollagen type 1 N-terminal propeptide; CTX: collagen type 1 cross-linked C-telopeptide; BALP: bone alkaline phosphatase; SePP: selenoprotein P; IOF: International Osteoporosis Foundation; IFCC: International Federation of Clinical Chemistry and Laboratory Medicine.

Supplemental Table 3: Estimated marginal means from ANCOVA of bone turnover markers by supplementation group at 6 months and 5 years after the removal of systemic glucocorticoid users. Upper and lower 95% confidence intervals are displayed in parentheses.

Bone Turnover (µg/L), Mean (CI)	Selenium dosage (µg)				P
	0	100	200	300	
OC 6 months	17.1 (16.3-17.9)	16.7 (16.0-17.5)	17.5 (16.7-18.4)	16.5 (15.7-17.4)	0.381
OC 5 years	17.0 (15.6-18.4)	17.2 (15.8-18.7)	17.2 (15.8-18.7)	16.1 (14.9-17.5)	0.642
PINP 6 months	38.7 (36.8-40.8)	36.6 (34.8-38.5)	38.4 (36.4-40.6)	38.7 (36.6-40.9)	0.394
PINP 5 years	39.7 (36.2-43.5)	40.0 (36.5-43.9)	39.4 (35.9-43.5)	37.7 (34.4-41.2)	0.801
CTX 6 months	0.16 (0.14-0.17)	0.16 (0.14-0.17)	0.16 (0.15-0.18)	0.15 (0.14-0.17)	0.916
CTX 5 years	0.16 (0.14-0.18)	0.17 (0.15-0.19)	0.16 (0.14-0.18)	0.15 (0.14-0.17)	0.621
BALP 6 months	14.4 (13.8-14.9)	13.8 (13.3-14.3)	13.7 (13.2-14.3)	14.3 (13.8-14.9)	0.192
BALP 5 years	14.0 (13.2-14.8)	14.9 (14.0-15.8)	14.5 (13.7-15.4)	14.6 (13.7-15.4)	0.526

CI: confidence intervals; OC: osteocalcin; PINP: procollagen type 1 N-terminal propeptide; CTX: collagen type 1 cross-linked C-telopeptide; BALP: bone alkaline phosphatase. Covariates in the ANCOVA included age (continuous), BMI (continuous), baseline selenium status (continuous), baseline BTM (continuous), smoking (binary), alcohol (binary), supplement use (binary) (calcium, vitamin D and multivitamins) and medication (binary) (thyroid, inhaled and systemic glucocorticoid (GC), antiresorptives, hormone replacement therapy (HRT)). OC 6 months n = 400: 105, 104, 98, 93; OC 5 years n = 325: 82, 83, 76, 84; PINP 6 months n = 399: 104, 104, 98, 93; PINP 5 years n = 325: 82, 83, 76, 84; CTX 6 months n = 374: 97, 97, 93, 87; CTX 5 years n = 296: 72, 76, 73, 75; BALP 6 months n = 398: 104, 103, 98, 93; BALP 5 years n = 325: 82, 82, 77, 84 participants for 0-300 µg/d selenium, respectively.

Supplemental Table 4: Estimated marginal means from ANCOVA of bone turnover markers by supplementation group at 6 months and 5 years after the removal of systemic glucocorticoid, inhaled glucocorticoid, thyroid medication and antiresorptive users. Upper and lower 95% confidence intervals are displayed in parentheses.

Bone Turnover (µg/L), Mean (CI)	Selenium dosage (µg)				P
	0	100	200	300	
OC 6 months	17.1 (16.3-17.9)	16.6 (15.6-17.4)	17.4 (16.6-18.3)	16.3 (15.5-17.1)	0.244
OC 5 years	16.9 (15.5-18.4)	16.9 (15.5-18.5)	17.1 (15.6-18.7)	15.9 (14.6-17.4)	0.689
PINP 6 months	38.9 (36.9-41.0)	36.4 (34.4-38.5)	38.2 (36.1-40.4)	38.4 (36.3-40.6)	0.346
PINP 5 years	39.0 (35.4-43.0)	39.9 (36.1-44.1)	39.4 (35.5-43.6)	37.5 (34.0-41.4)	0.847
CTX 6 months	0.16 (0.14-0.17)	0.15 (0.14-0.16)	0.16 (0.15-0.18)	0.15 (0.14-0.17)	0.715
CTX 5 years	0.17 (0.15-0.19)	0.17 (0.15-0.19)	0.16 (0.14-0.18)	0.16 (0.14-0.18)	0.815
BALP 6 months	14.4 (13.9-15.0)	13.7 (13.2-14.2)	13.7 (1.32-14.3)	14.2 (13.6-14.7)	0.174
BALP 5 years	14.0 (13.2-14.8)	14.7 (13.8-15.7)	14.5 (13.6-15.5)	14.4 (13.5-15.3)	0.673

CI: confidence intervals; OC: osteocalcin; PINP: procollagen type 1 N-terminal propeptide; CTX: collagen type 1 cross-linked C-telopeptide; BALP: bone alkaline phosphatase. Covariates in the ANCOVA included age (continuous), BMI (continuous), baseline selenium status (continuous), baseline BTM (continuous), smoking (binary), alcohol (binary), supplement use (binary) (calcium, vitamin D and multivitamins) and medication (binary) (thyroid, inhaled and systemic glucocorticoid (GC), antiresorptives, hormone replacement therapy (HRT)). OC 6 months n = 371: 101, 92, 90, 88; OC 5 years n = 296: 78, 73, 69, 76; PINP 6 months n = 370: 100, 92, 90, 88; PINP 5 years n = 296: 78, 73, 69, 76; CTX 6 months n = 347: 94, 86, 85, 82; CTX 5 years n = 269: 69, 67, 66, 67; BALP 6 months n = 369: 100, 91, 90, 88; BALP 5 years n = 296: 78, 72, 70, 76 participants for 0-300 µg/d selenium, respectively.

Supplemental Table 5: Estimated marginal means from ANCOVA of bone turnover markers by supplementation group at 6 months and 5 years after the removal of hormone replacement therapy users. Upper and lower 95% confidence intervals are displayed in parentheses.

**Bone Turnover (µg/L), Mean (CI)**	**Selenium dosage (µg)**				**P**
	**0**	**100**	**200**	**300**	
**OC 6 months**	17.4 (16.6-18.2)	17.3 (16.5-18.2)	17.5 (16.6-18.4)	16.7 (16.0-17.6)	0.649
**OC 5 years**	17.4 (15.9-19.0)	17.3 (15.8-18.9)	17.5 (16.0-18.9)	16.1 (14.7-17.5)	0.505
**PINP 6 months**	38.7 (36.6-40.9)	37.9 (35.9-40.2)	39.4 (37.2-41.6)	40.1 (37.8-42.5)	0.605
**PINP 5 years**	40.9 (36.9-45.3)	41.1 (37.1-45.5)	40.9 (36.9-45.3)	38.1 (34.4-42.1)	0.684
**CTX 6 months**	0.16 (0.14-0.17)	0.16 (0.14-0.18)	0.16 (0.15-0.18)	0.16 (0.14-0.17)	0.846
**CTX 5 years**	0.16 (0.14-0.18)	0.17 (0.15-0.20)	0.16 (0.14-0.19)	0.15 (0.13-0.17)	0.523
**BALP 6 months**	14.6 (14.0-15.2)	14.0 (13.2-14.6)	13.7 (13.2-14.3)	14.5 (13.9-15.1)	0.149
**BALP 5 years**	14.4 (13.5-15.3)	15.1 (14.1-16.1)	14.9 (14.0-16.0)	14.6 (13.7-15.6)	0.737

CI: confidence intervals; OC: osteocalcin; PINP: procollagen type 1 N-terminal propeptide; CTX: collagen type 1 cross-linked C-telopeptide; BALP: bone alkaline phosphatase. Covariates in the ANCOVA included age (continuous), BMI (continuous), baseline selenium status (continuous), baseline BTM (continuous), smoking (binary), alcohol (binary), supplement use (binary) (calcium, vitamin D and multivitamins) and medication (binary) (thyroid, inhaled and systemic glucocorticoid (GC), antiresorptives, hormone replacement therapy (HRT)). OC 6 months n = 341: 87, 87, 86, 81; OC 5 years n = 296: 78, 73, 69, 76; PINP 6 months n = 340: 86, 87, 86, 81; PINP 5 years n = 268: 66, 67, 66, 69; CTX 6 months n = 321: 81, 82, 81, 77; CTX 5 years n = 244: 59, 61, 63, 61; BALP 6 months n = 339: 86, 86, 86, 81; BALP 5 years n = 269: 66, 67, 67, 69 participants for 0-300 µg/d selenium, respectively.

Supplemental Table 6: Estimated marginal means from ANCOVA of bone turnover markers by supplementation group at 6 months and 5 years after the removal of calcium, vitamin D and multivitamin users. Upper and lower 95% confidence intervals are displayed in parentheses.

**Bone Turnover (µg/L), Mean (CI)**	**Selenium dosage (µg)**				**P**
	**0**	**100**	**200**	**300**	
**OC 6 months**	17.1 (16.1-18.0)	16.8 (15.8-17.8)	17.6 (16.4-18.7)	17.1 (16.0-18.2)	0.785
**OC 5 years**	16.7 (15.1-18.5)	17.9 (16.2-19.9)	16.7 (14.7-18.9)	16.4 (14.7-18.4)	0.671
**PINP 6 months**	38.3 (35.9-40.8)	35.8 (33.6-38.2)	38.0 (35.3-40.9)	39.5 (36.9-42.5)	0.226
**PINP 5 years**	38.6 (34.4-43.4)	41.2 (36.6-46.2)	37.5 (32.6-43.2)	38.2 (33.7-43.4)	0.743
**CTX 6 months**	0.17 (0.15-0.19)	0.16 (0.14-0.17)	0.16 (0.14-0.18)	0.16 (0.14-0.18)	0.879
**CTX 5 years**	0.18 (0.15-0.21)	0.18 (0.16-0.21)	0.16 (0.14-0.19)	0.17 (0.14-0.19)	0.589
**BALP 6 months**	14.1 (13.6-14.7)	13.6 (13.1-14.2)	13.8 (13.2-14.4)	14.5 (13.9-15.2)	0.138
**BALP 5 years**	14.1 (13.1-15.2)	15.2 (14.1-16.4)	14.2 (13.0-15.5)	14.3 (13.2-15.4)	0.488

CI: confidence intervals; OC: osteocalcin; PINP: procollagen type 1 N-terminal propeptide; CTX: collagen type 1 cross-linked C-telopeptide; BALP: bone alkaline phosphatase. Covariates in the ANCOVA included age (continuous), BMI (continuous), baseline selenium status (continuous), baseline BTM (continuous), smoking (binary), alcohol (binary), supplement use (binary) (calcium, vitamin D and multivitamins) and medication (binary) (thyroid, inhaled and systemic glucocorticoid (GC), antiresorptives, hormone replacement therapy (HRT)). OC 6 months n = 246: 70, 69, 50, 57; OC 5 years n = 199: 57, 57, 37, 48; PINP 6 months n = 245: 69, 69, 50, 57; PINP 5 years n = 200: 57, 57, 38, 48; CTX 6 months n = 232: 67, 64, 47, 54; CTX 5 years n = 179: 50, 52, 35, 42; BALP 6 months n = 244: 69, 68, 50, 57; BALP 5 years n = 199: 57, 56, 38, 48 participants for 0-300 µg/d selenium, respectively.

Supplemental Table 7: Estimated marginal means from ANCOVA of bone turnover markers by supplementation group at 6 months and 5 years after the removal of antiresorptive users and fractures. Upper and lower 95% confidence intervals are displayed in parentheses.

**Bone Turnover (µg/L), Mean (CI)**	**Selenium dosage (µg)**				**P**
	**0**	**100**	**200**	**300**	
**OC 6 months**	17.1 (16.3-17.9)	16.7 (15.9-17.5)	17.4 (16.6-18.3)	16.5 (15.7-17.3)	0.405
**OC 5 years**	16.8 (15.5-18.2)	17.3 (15.9-18.8)	17.1 (15.7-18.6)	16.0 (14.8-17.4)	0.602
**PINP 6 months**	38.9 (36.9-41.0)	36.6 (34.7-38.5)	38.4 (36.3-40.6)	38.5 (36.5-40.8)	0.373
**PINP 5 years**	39.4 (36.0-43.3)	40.6 (36.9-44.6)	39.4 (35.7-43.4)	37.3 (34.0-40.9)	0.658
**CTX 6 months**	0.16 (0.14-0.17)	0.16 (0.15-0.18)	0.16 (0.15-0.18)	0.16 (0.14-0.17)	0.914
**CTX 5 years**	0.16 (0.14-0.18)	0.18 (0.16-0.20)	0.16 (0.14-0.18)	0.15 (0.14-0.17)	0.447
**BALP 6 months**	14.4 (13.8-14.9)	13.8 (13.2-14.3)	13.7 (13.2-14.2)	14.3 (13.7-14.9)	0.183
**BALP 5 years**	13.9 (13.1-14.7)	13.1 (14.2-15.9)	14.5 (13.6-15.3)	14.4 (13.6-14.4)	0.316

CI: confidence intervals; OC: osteocalcin; PINP: procollagen type 1 N-terminal propeptide; CTX: collagen type 1 cross-linked C-telopeptide; BALP: bone alkaline phosphatase. Covariates in the ANCOVA included age (continuous), BMI (continuous), baseline selenium status (continuous), baseline BTM (continuous), smoking (binary), alcohol (binary), supplement use (binary) (calcium, vitamin D and multivitamins) and medication (binary) (thyroid, inhaled and systemic glucocorticoid (GC), antiresorptives, hormone replacement therapy (HRT)). OC 6 months n = 392: 104, 100, 97, 91; OC 5 years n = 316: 81, 79, 75, 81; PINP 6 months n = 391: 103, 100, 97, 91; PINP 5 years n = 316: 81, 79, 75, 81; CTX 6 months n = 366: 96, 93, 92, 85; CTX 5 years n = 289: 71, 73, 72, 73; BALP 6 months n = 390: 103, 99, 97, 91; BALP 5 years n = 316: 81, 78, 76, 81 participants for 0-300 µg/d selenium, respectively.

Supplemental Table 8: Sensivity analyses including participants with plasma selenium status below 70 µg/L at baseline. Upper and lower 95% confidence intervals are displayed in parentheses.

**Bone Turnover (µg/L), Mean (CI)**	**Selenium dosage (µg)**				**P**
	**0**	**100**	**200**	**300**	
**OC 6 months**	16.4 (14.3-18.7)	16.8 (14.7-19.3)	18.8 (16.4-21.5)	15.7 (14.2-17.3)	0.231
**OC 5 years**	25.6 (18.3-35.7)	14.5 (10.2-20.6)	16.1 (10.5-24.7)	18.7 (14.8-23.5)	0.139
**PINP 6 months**	37.3 (30.5-45.6)	37.6 (30.5-46.3)	40.6 (33.3-49.4)	38.8 (33.5-44.9)	0.930
**PINP 5 years**	56.5 (40.4-78.9)	37.1 (26.2-52.4)	37.1 (24.2-56.6)	43.6 (34.8-54.6)	0.318
**CTX 6 months**	0.17 (0.12-0.24)	0.15 (0.10-0.22)	0.15 (0.10-0.21)	0.15 (0.11-0.19)	0.931
**CTX 5 years**	0.27 (0.19-0.38)	0.16 (0.11-0.25)	0.12 (0.10-0.19)	0.16 (0.12-0.20)	0.045
**BALP 6 months**	13.5 (11.8-15.6)	12.9 (11.3-14.7)	13.5 (11.8-15.3)	14.4 (13.1-15.8)	0.580
**BALP 5 years**	16.5 (12.4-21.9)	13.5 (10.0-18.1)	13.2 (9.9-18.7)	14.9 (12.3-17.9)	0.728

CI: confidence intervals; OC: osteocalcin; PINP: procollagen type 1 N-terminal propeptide; CTX: collagen type 1 cross-linked C-telopeptide; BALP: bone alkaline phosphatase. Covariates in the ANCOVA included age (continuous), BMI (continuous), baseline selenium status (continuous), baseline BTM (continuous), smoking (binary), alcohol (binary), supplement use (binary) (calcium, vitamin D and multivitamins) and medication (binary) (thyroid, inhaled and systemic glucocorticoid (GC), antiresorptives, hormone replacement therapy (HRT)). OC 6 months n = 49: 10, 9, 11, 19; OC 5 years n = 38: 8, 7, 6, 17; PINP 6 months n = 49: 10, 9, 11, 19; PINP 5 years n = 38: 8, 7, 6, 17; CTX 6 months n = 44: 9, 7, 10, 18; CTX 5 years n = 35: 8, 6, 6, 15; BALP 6 months n = 48:9, 9, 11, 19; BALP 5 years n = 38: 8, 7, 6, 17 participants for 0-300 µg/d selenium, respectively.

Supplemental Table 9: Baseline characteristics of participants with bone turnover markers by dropout status (yes/no).

Characteristic	All Participants	Dropout		P
		No	Yes	
**Sex, n (%) Male**	250 (52.0)	177 (50.0)	73 (57.5)	0.148
**Sex, n (%) Female**	231 (48.0)	177 (50.0)	54 (42.5)	
**Age years, Mean (SD)**	66.16 (4.1)	66.10 (4.1)	66.34 (4.2)	0.571
**Plasma Selenium µg/L, Mean (SD)**	86.54 (16.2)	86.71 (16.4)	85.89 (15.8)	0.742
**BMI kg/m^2, Mean (SD)**	26.82 (4.03)	26.81 (4.0)	26.88 (4.0)	0.672
**Height m, Mean (SD)**	1.69 (0.09)	1.69 (0.09)	1.69 (0.09)	0.719
**Weight kg, Mean (SD)**	76.81 (13.6)	76.76 (13.6)	77.17 (13.2)	0.720
**Alcohol units per week, Mean (SD)**	7.29 (7.5)	7.27 (7.6)	7.39 (7.4)	0.865
**Smokers, n (%)**				0.220
Never	158 (32.8)	120 (33.9)	38 (29.9)	
Previous	178 (37.0)	135 (38.1)	43 (33.9)	
Present	145 (30.1)	99 (28.0)	46 (36.2)	
**Education, n (%)**				0.397
None	134 (28.7)	103 (29.7)	31 (25.8)	
1-3y	76 (16.3)	53 (15.3)	23 (19.2)	
3-4y	212 (45.4)	161 (46.4)	51 (42.5)	
>4y	45 (9.6)	30 (8.6)	15 (12.5)	
**Live Alone, n (%)**				0.713
No	400 (85.7)	296 (85.3)	104 (86.7)	
Yes	67 (14.3)	51 (14.7)	16 (13.3)	
**Thyroid Medication, n (%)**				0.961
None	468 (97.1)	344 (97.2)	123 (96.9)	
LT4	11 (2.3)	8 (2.3)	3 (2.4)	
ATD	3 (0.6)	2 (0.6)	1 (0.8)	
**Inhaled GC, n (%)**				0.721
No	451 (96.4)	335 (96.5)	115 (95.8)	
Yes	17 (3.6)	12 (3.5)	5 (4.2)	
**Systemic GC, n (%)**				0.274
No	475 (99.2)	352 (99.4)	123 (98.4)	
Yes	4 (0.8)	2 (0.6)	2 (1.6)	
**Antiresorptives, n (%)**				0.667
No	461 (98.7)	343 (98.8)	118 (98.3)	
Yes	6 (1.3)	4 (1.2)	2 (1.7)	
**HRT, n (%)**				0.128
No	392 (83.9)	286 (82.4)	106 (88.3)	
Yes	75 (16.1)	61 (17.6)	14 (11.7)	
**Calcium, n (%)**				0.642
No	419(89.7)	310 (89.3)	109 (90.8)	
Yes	48 (10.3)	37 (10.7)	11 (9.2)	
**Vitamin D, n (%)**				0.254

No	442 (94.6)	326 (93.9)	116 (96.7)	
Yes	25 (5.4)	21 (6.1)	4 (3.3)	
**Multivitamin, n (%)**				0.911
No	325 (69.6)	241 (69.5)	84 (70.0)	
Yes	142 (30.4)	106 (30.5)	36 (30.0)	
**Dosage, n (%)**				0.847
0	124 (25.8)	88 (24.9)	36 (28.3)	
100	122 (25.3)	90 (25.4)	32 (25.2)	
200	118 (24.5)	87 (24.6)	31 (24.4)	
300	117 (24.3)	89 (25.1)	28 (22.0)	

SD: standard deviation; inhaled GC: inhaled glucocorticoid; systemic GC: systemic glucocorticoid; LT4: levothyroxine; ATD: antithyroid drugs; HRT: hormone replacement therapy. Age, alcohol, smokers n = 482; weight, height, BMI n = 479; plasma selenium baseline n = 479.

Supplemental Table 10: Bone turnover markers at baseline of participants by dropout status (yes/no).

**Bone Turnover (μg/L), Mean (SD)**	**All participants**	**Dropout**		**P**
		**No**	**Yes**	
**OC**	18.67 (8.46)	18.60 (8.23)	18.86 (9.09)	0.883
**PINP**	42.68 (18.05)	42.14 (17.23)	44.17 (20.16)	0.520
**CTX**	0.20 (0.22)	0.21 (0.25)	0.20 (0.13)	0.876
**BALP**	15.66 (5.68)	15.60 (5.83)	15.84 (5.24)	0.279

SD: standard deviation; OC: osteocalcin; PINP: procollagen type 1 N-terminal propeptide; CTX: collagen type 1 cross-linked C-telopeptide; BALP: bone alkaline phosphatase. OC and PINP n = 482; CTX n = 460; BALP n = 480. OC and PINP n = 354 remained, 127 dropped out; CTX n = 336 remained, 123 dropped out; BALP n = 354 remained, 125 dropped out.
